# Andrographolide inhibits the upregulation of SLC19A3 to block the adipogenic differentiation of adipose-derived stem cells

**DOI:** 10.1016/j.isci.2025.114379

**Published:** 2025-12-08

**Authors:** Yili Yang, Xuan Wang, Guangfeng Zhou, Xiaodan Hou, Yang He, Yuan Feng, Yuxue Jiang, Yuexi Gu, Jun Ye

**Affiliations:** 1 China Regional Research Centre, International Centre for Genetic Engineering and Biotechnology (ICGEB), Taizhou, Jiangsu 225316, P.R. China; 2 Center for Public Health Research, School of Medicine, Nanjing University, Nanjing, Jiangsu 211166, P.R. China; 3 Suzhou Kintor Pharmaceuticals, Suzhou, Jiangsu 215127, P.R. China; 4 Helixon Biotechnology, Suzhou, Jiangsu 215128, P.R. China; 5 Suzhou Institute of Systems Medicine, Center for Systems Medicine, Chinese Academy of Medical Sciences, Suzhou, Jiangsu 215004, P.R. China; 6 Central Laboratory, The Affiliated Taizhou People’s Hospital of Nanjing Medical University, Taizhou School of Clinical Medicine, Nanjing Medical University, Taizhou, Jiangsu 225300, P.R. China

**Keywords:** Biological sciences, Cell biology, Health sciences, Natural product biochemistry, Natural sciences

## Abstract

We found that andrographolide (AP), the main active component of medicinal herb *Andrographis paniculata*, inhibited the differentiation of adipose-derived stem cells (ADSCs) toward adipocytes in culture and in mice fed with high fat diet. RNA-seq analyses revealed that the induction of SLC19A3, a thiamine transporter, was blocked by AP, and knockdown of SLC19A3 abolished the adipogenic differentiation. Further, supplementing thiamine to cultured ADSCs reversed the inhibitory effect of AP dose-dependently, indicating that SLC19A3 mediates the action of AP on adipogenic differentiation. Interestingly, the differentiation of ADSCs was accompanied by the reduction of S9-phosphorylated GSK3β, which was prevented by AP. Subsequent investigation showed that AP and a GSK3β inhibitor upregulated Snail1, a transcriptional repressor influencing cell differentiation. While enforced expression of Snail1 reduced the luciferase activity of the SLC19A3 reporter, siRNA against Snail1 blocked the inhibition of AP on SLC19A3 expression. Thus, the GSK3β-Snail1-SLC19A3 axis mediates the anti-adipogenic action of andrographolide.

## Highlights

• Andrographolide (AP) inhibits the differentiation of ADSCs toward adipocytes
• AP prevents the development of obesity in a high-fat diet (HFD)-induced mouse model
• The anti-adipogenic and anti-obese effects of AP are dependent on its inhibition of GSK3β
• The effects of AP are mediated by Snail1, which downregulates SLC19A3 upon GSK3β inhibition

## Graphical abstract

**Figure:**
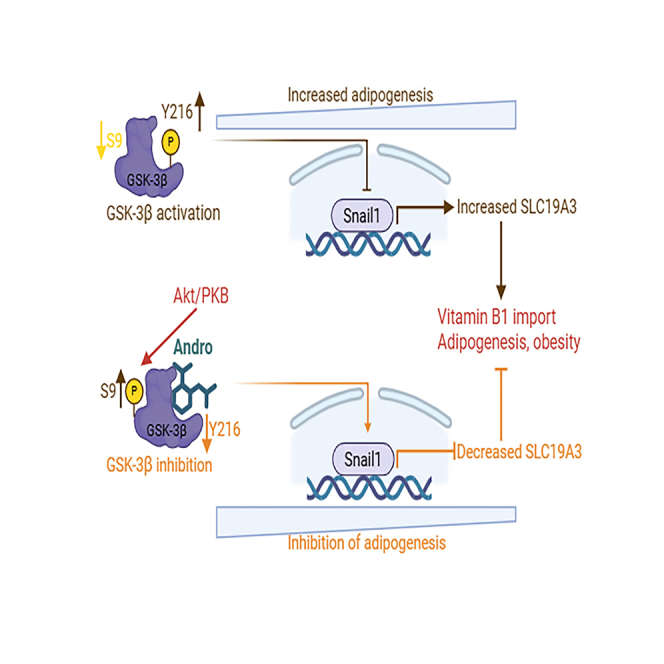

## Introduction

*Andrographis paniculata*, an annual herbaceous plant in the family of Acanthaceae, has been used in traditional medicines of India, China, and South Asia for centuries. One of its major active components is andrographolide (AP), a diterpene lactone possessing a variety of pharmacological activities, including antioxidant, anti-inflammatory, anti-cancer, and immuno-modulation.^1^^,^^2^ AP has also been used in clinical trials as an effective anti-inflammatory drug with a relatively safe record.^3^^,^^4^ To further increase its selectivity and bio-availability, efforts have been devoted in many laboratories to modify its structure and generate multiple analogs.^5^^,^^6^ It has been reported that some of the analogs are more potent anti-cancer agents, and others are effective in inhibiting inflammation without suppressing the innate immunity, indicating that they are potential novel therapeutic agents.^6^^,^^7^ However, the molecular mechanisms of the multiple actions of AP are still not comprehensively understood, although a number of molecules, including NF-κB, PI3K, NFAT, caspase-1 and Nrf2, have been implied under various experimental conditions.^8^^,^^9^

Obesity has been recognized as a global epidemic since the end of last century. Excessive adipose tissue accumulation is regarded as the result of both increased numbers and sizes of mature adipocytes.^10^^,^^11^ It has been shown that increased differentiation of the precursors of adipocytes, often designated as adipose-derived stem cells (ADSCs), plays a critical role in the increase of adipocytes in obese individuals.^12^^,^^13^ ADSCs are phenotypically CD34^−^/CD45^−^/CD73^+^/CD105^+^.^14^^,^^15^ *In vivo*, their differentiation is regulated mainly by insulin-like growth factor 1 (IGF-1), transforming growth factor-beta (TGFβ), and fibroblast growth factors (FGFs).^16^^,^^17^ In culture, they may differentiate into adipocytes under the coordinated actions of dexamethasone, indomethacin and insulin.^18^^,^^19^ In addition to the morphological changes, well-known molecular markers for the differentiation of ADSCs include C/EBPα, PPARγ, FABP4, and LPL.^20^^,^^21^ Of note, ADSCs are also regarded as a promising type of mesenchymal stem cells for their immuno-modulatory actions in the treatment of autoimmune diseases, and as an abundant cell source for tissue engineering.^22^^,^^23^

In an effort to find natural compounds that affect the differentiation of ADSCs, we screened a natural product library and found that AP effectively inhibited the differentiation in culture. It also reduced the weight of fat tissues in mice fed with a high-fat diet (HFD). Through RNA-seq analysis, we found that AP significantly blocked the induction of SLC19A3 during adipogenic differentiation. Of note, SLC19A3 encodes a thiamine (vitamin B1) transporter and is highly expressed in fat tissues.^24^^,^^25^ While SLC19A3 knockdown prevented the adipogenic differentiation, supplementation of thiamine to cultured ADSCs abolished the inhibitory effects of AP, indicating that the anti-differentiation action of AP is mediated by the downregulation of SLC19A3 and subsequent reduced uptake of thiamine. Further analyses revealed that the S9-phosphorylated inactive GSK3β and its substrate β-catenin are reduced during the differentiation of ADSCs, whereas AP effectively prevented those alterations. We also found that AP and inhibition of GSK3β with a small molecular inhibitor upregulated the levels of transcriptional repressor Snail1, which plays an important role in gene expression and cell differentiation.^26^ Knocking down of Snail1 diminished the reduction of SLC19A3 induced by AP, whereas enforced expression of Snail1 suppressed the luciferase activity by a reporter containing the upstream sequence of SLC19A3. Thus, it is evident that the GSK3β-Snail1-SLC19A3 axis mediates the inhibitory action of AP on adipogenesis. It is also conceivable that AP might be used as ADSCsa potential therapeutic agent for the management of obesity and related diseases.

## Results

### AP inhibits the differentiation of adipose-derived stem cells toward adipocytes

**Figure 1:**
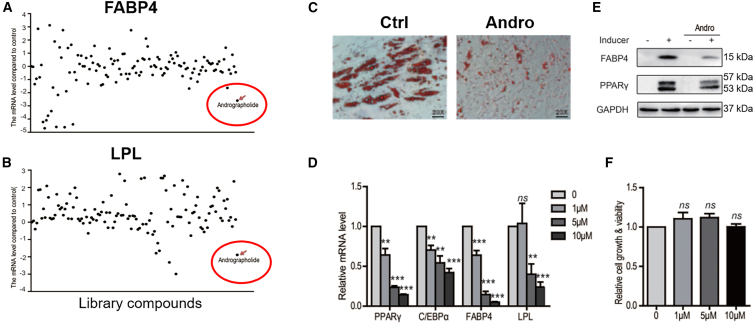
Andrographolide inhibits the adipogenic differentiation of ADSCs (A and B) screening for natural products that inhibit FABP4 (A) and LPL (B) expression. (C) Andrographolide prevents the formation of adipocytes. Scale bars, 100 μm. (D) Andrographolide downregulated the expression differentiation markers dose-dependently. (E) Andrographolide abolished the upregulation of FABP4 and PPARγ protein expression. (F) Andrographolide did not affect the growth of ADSCs as measured by CCK-8. Data are expressed as mean ± SEM. ^∗∗^*p* < 0.01, ^∗∗∗^*p* < 0.001.

ADSCs, which express mesenchymal stem cell markers CD73 and CD105 and were negative for the hematopoietic stem cell marker CD34 (Supplement Figure 1A), had a spindle-shaped fibroblast-like morphology and formed a flattened monolayer in our culture. After cultured in the induction medium for ∼3 days, these cells differentiated and expressed adipogenic markers, including C/EBPα, PPARγ, FABP4, and LPL. By day 10–14, they became large round-shaped and generated lipid droplets that were stained by oil red O, indicative of the formation of adipocytes (Figures S1B–S1D). In an effort to find natural compounds that regulate the adipogenic differentiation of ADSCs, we screened a natural products library of 124 compounds for inhibiting the expressions of LPL and FABP4 (Table S1). As shown in Figure 1A, andrographolide (AP), a main bioactive component of the herbal plant *Andrographis paniculata*, was found to be the only chemical that inhibited the expressions of both LPL and FABP4 expression. We then examined the effects of AP on more markers and cell differentiation. It inhibited the upregulation of C/EBPα, PPARγ, FABP4, and LPL dose-dependently in ADSCs exposed to the induction medium by RT-qPCR and immunoblotting (Figures 1D and 1E). Further, AP blocked the formation of oil droplets stained by oil red O in these cells, without significantly affecting the cell viability (Figures 1C and 1F).

### AP reduces the increase of body fat in HFD-fed mice

**Figure 2:**
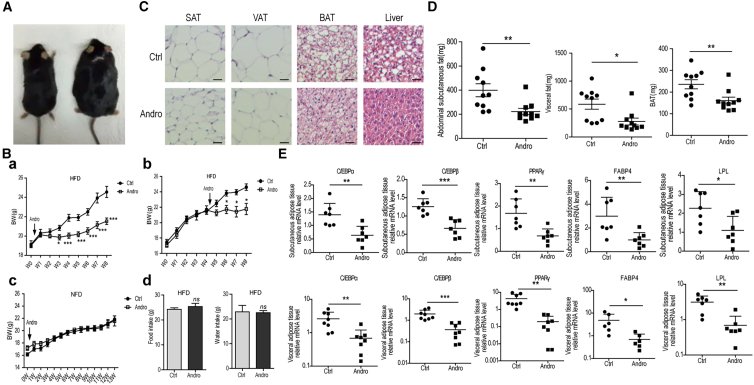
Andrographolide prevents HFD-induced weight increase and adipogenic differentiation (A and B) Body weights of mice fed with HFD increased markedly. Intragastric administration of andrographolide reduced the increase significantly. AP does not change body weight of mice received normal diet, and did not affect the amounts of the food intake. (C) Histology of mice fed with HFD with or without given AP. Scale bars, 100 μm. (D) Weights of visceral, subcutaneous, and brown adipose tissues from HFD- and HFD plus andrographolide-fed mice. (E) Expression of differentiation markers CEBPα, CEBPβ, PPARγ, FABP4, and LPL in visceral, subcutaneous, and brown adipose tissues from HFD- and HFD plus andrographolide-fed mice. Data are expressed as mean ± SEM. ^∗^*p* < 0.05, ^∗∗^*p* < 0.01, ^∗∗∗^*p* < 0.001.

To determine if the effect of AP on cultured ADSCs reflects its activity *in vivo*, we administered AP to mice fed with the HFD. As shown in Figure 2A, C57BL/6 mice received HFD had markedly increased body size compared to those given regular diet. When administered intra-gastrically to mice at the same time of feeding the HFD, AP decreased significantly the body weight increase after week 2 (Figure 2B a). Moreover, when AP was given to mice that had been fed with the HFD for 4 weeks, it remained effective in reducing the increase of body weight (Figure 2B b). Of note, AP did not affect the body weight in mice fed with normal diet (Figure 2B c), and it did not decrease food and water intake in mice taken HFD either (Figure 2B d). We then examined HE-stained sections of subcutaneous, visceral, and brown adipose tissues, and liver from HFD-fed mice and those additionally administered with AP. As shown in Figure 2C, AP reduced the size of fat cells in all types of adipose tissues, and decreased the amount of fat in liver cells.

To determine whether the fat reduction induced by AP is related to the ADSCs differentiation, we collected the subcutaneous, visceral, and brown adipose tissues and examined the levels of the differentiation markers C/EBPα, PPARγ, FABP4, and LPL in HFD-fed mice with or without AP administration. It was found that the fat tissues collected from mice fed with HFD and AP weighted significantly lower than those fed with HFD only (Figure 2D). The mRNA levels of C/EBPα, PPARγ, FABP4, and LPL were then quantitated by RT-qPCR. As show in Figure 2E, despite variations in individual mice, there were significant decreases in all the markers examined in mice fed HFD and AP in comparison with these given HFD only. Of note, AP also decreased the production of inflammatory cytokines associated with HFD-induced obesity (Figure S2). These results indicate that there are close connections between the AP-induced ADSC differentiation and reduction of body and adipose tissue weights.

### AP inhibits expression of the thiamine transporter SLC19A3

**Figure 3:**
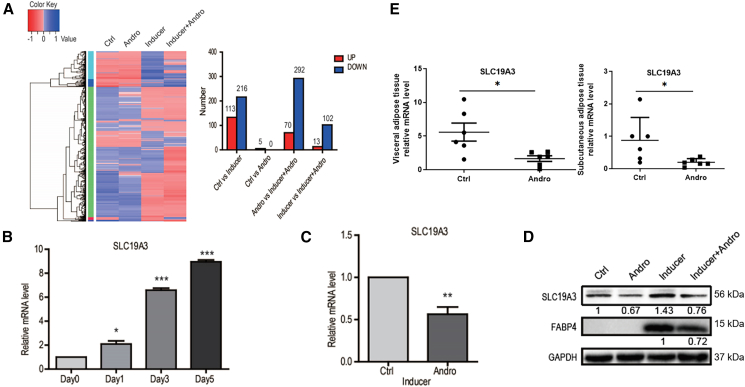
Andrographolide (AP) inhibits the upregulation of thiamine (vitamin B1) transporter SLC19A3 in ADSCs and in fat tissues from mice fed with HFD (A) RNA-seq analysis of ADSCs induced to adipogenic differentiation in the presence and absence of AP. (B) Induction of SLC19A3 during the differentiation of ADSCs (*n* = 3). (C) AP prevented the upregulation of SLC19A3 mRNA (*n* = 3). (D) AP diminished the increase of SLC19A3 and FABP4 proteins. The numbers under each band represent relative density compared to the controls. (E) Expression of SLC19A3 in visceral and subcutaneous fat tissues from mice fed with HFD or HFD plus AP. Data are expressed as mean ± SEM. ^∗^*p* < 0.05, ^∗∗^*p* < 0.01, ^∗∗∗^*p* < 0.001.

To understand the mechanisms by which AP inhibits their differentiation, ADSCs were cultured in control or adipogenic-inducing medium in the absence or presence of 10 μM AP for 7 days. Total RNAs were then extracted for RNA-seq analysis to identify genes whose expressions were altered. While AP alone had relatively mild effects on gene expression in cells cultured in control medium, it changed the gene expression profile of ADSCs grown in the induction medium markedly (Figure 3A), suggesting that AP exerted its effects mainly on the differentiation process but did not affect the stem cell populations. In fact, ADSCs treated with the inducing medium and AP together resulted in the upregulation of only 13 genes, but downregulation of 102 genes, when compared with the cells exposed to the induction medium alone (Figure 3A).

**Figure 4:**
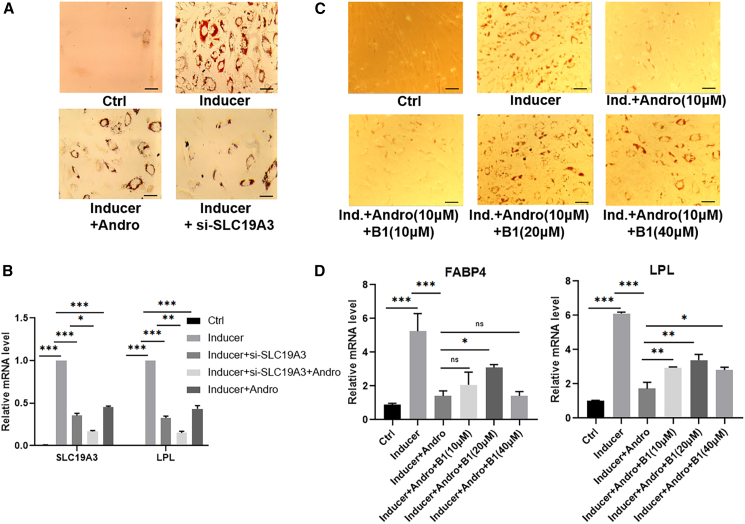
SLC19A3 mediates the adipogenic differentiation-inhibiting action of andrographolide (A and B) Knock-down of SLC19A3 blocks the adipogenic differentiation of ADSCs examined by oil red O staining (Scale bars, 100 μm) (A), SLC19A3 and LPL expression (B). (C and D) Thiamine reversed the effect of andrographolide on AdSC differentiation (Scale bars, 100 μm) (C) and markers expression (D). Data are expressed as mean ± SEM. ^∗^*p* < 0.05, ^∗∗^*p* < 0.01, ^∗∗∗^*p* < 0.001.

Interestingly, SLC19A3, a member of the solute carrier family that transports thiamine (Vitamin B1),^24^ was one of the genes downregulated most significantly (∼7-fold). Its expression was also reduced in HFD-fed mice given AP (Figure 3E). Further analyses found that, during the differentiation of ADSCs, SLC19A3 was induced gradually and reached the highest level at day 7 (Figure 3B). As shown in Figures 3C and 3D, AP inhibited the upregulation of SLC19A3 mRNA and protein effectively in ADSCs. To determine whether downregulation of SLC19A3 is required for the inhibitory action of AP on ADSCs differentiation, we applied siRNA against SLC19A3. Like AP, transfection of siRNA decreased the level of cellular SLC19A3, inhibited the expression of differentiation markers FABP4 and LPL, and blocked the formation of lipid droplets in these cells (Figures 4A and 4B). Moreover, the actions of AP and the siRNA were additive, supporting the notion that AP exerts its inhibitory action on adipogenic differentiation through preventing the upregulation of SLC19A3.

To further investigate whether down-regulation of SLC19A3 is sufficient for the inhibitory effects of AP on ADSCs differentiation, we supplemented indicated amounts of thiamine in the culture medium to see whether it would affect the action of AP. As shown in Figure 3C, thiamine reversed the inhibitory action of AP lipid drop formation and cell differentiation dose-dependently. It also released the inhibition on differentiation markers PPARγ, FABP4, and SLC19A3 mildly (Figure 4D), presumably due to the initiation of the differentiation program. These results indicated that down regulation of SLC19A3 and subsequent reduction of thiamine transportation is at least partially mediated the anti-differentiation action of AP.

### AP affects Wnt/β-catenin signaling pathway by inhibiting GSK3beta activity

**Figure 5:**
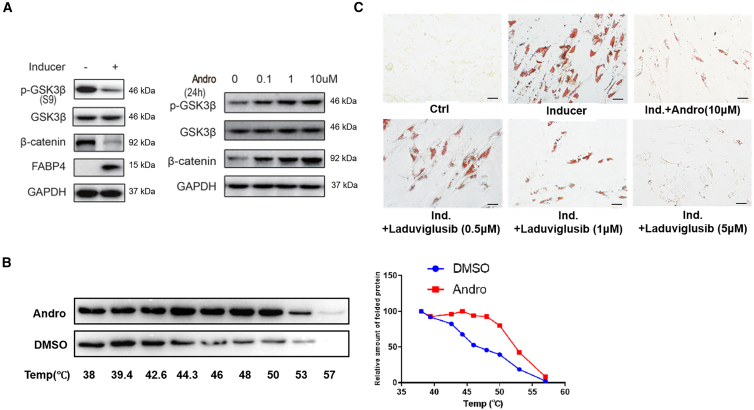
Andrographolide acts on GSK3β (A) GSK3β was dephosphorylated at S9 upon inducing differentiation of ADSCs toward adipocytes. AP prevents the dephosphorylation of GSK3β dose-dependently. (B) In cellular thermal shift assay, AP protected GSK3β from denaturing and increases the amount of protein in the supernatants, indicative of direct binding. (C) Similar to AP, small molecular inhibitor of GSK3β laduviglusib also blocks the adipogenic differentiation of ADSCs as revealed by oil red O staining. Scale bars, 100 μm.

Wnt/β-catenin signaling is critical for maintaining the stemness of stem cells,^27^ and has been shown to affect adipogenesis.^28^^,^^29^ Therefore, we sought to examine the status of GSK3β and β-catenin in the process of ADSCs differentiation. While the ADSCs in normal medium had high levels of phosphorylated GSK3β at S9 and β-catenin, exposure to the induction medium for 3 days resulted in reduced level of inactive S9-phosphorylated GSK3β, decrease of β-catenin, and expression of differentiation marker FABP4 (Figure 5A). Interestingly, AP prevented the reduction of S9-phosphorylated GSK3β in a dose-dependent manner (Figure 5A), leading to increased β-catenin that blocks ADSCs differentiation.

Cellular thermal shift assay (CETSA) is an effective method for discovering and evaluating the target proteins of drugs in cells and tissue samples.^30^ In our experiments, ADSCs were exposed to 10 mM of AP and heated for the indicated time points. Following cell lysis through freeze-thaw cycles, the cell lysates were analyzed using immunoblotting with an anti-GSK3β antibody. As shown in Figure 5B, the amount of soluble GSK3β started to decrease at 44°C in the absence of AP, whereas, in the presence of AP, it only showed an apparent reduction at ∼50°C, indicating stability of GSK3β was markedly increased by AP. Therefore, AP likely acts on GSK3β.

Those results propelled us to examine the effects of small molecular inhibitor of GSK3β on the differentiation of ADSCs and expression of SLC19A3. Indeed, the GSK3β inhibitor laduviglusib co-culture with ADSCs blocked upregulation of adipogenic differentiation markers FABP4 and LPL, and prevented the formation of lipid droplets in the cells dose-dependently (S3). Similar to AP, it also inhibited the expression of SLC19A3 effectively. These results confirm the notion that AP-led inhibition of GSK3β is critical for downregulated SLC19A3 and prevention of ADSCs differentiation.

### Snail1 mediates the regulation of GSK3β inhibition on SLC19A3

**Figure 6:**
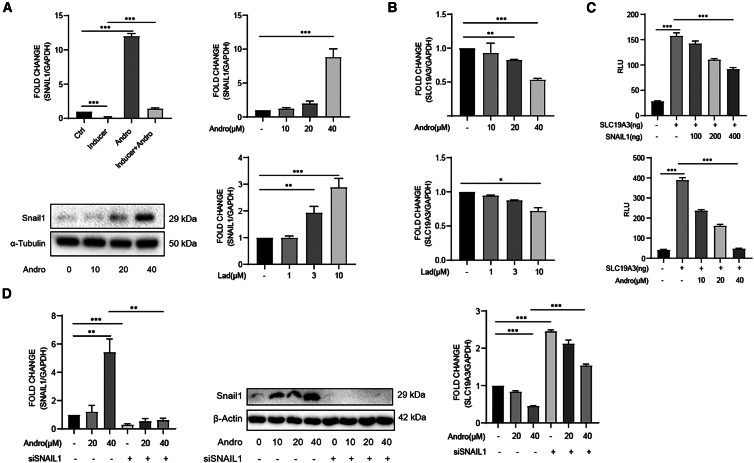
Snail1 mediates the regulation of GSK3β inhibition on SLC19A3 (A) The expression of Snail1 is increased in ADSCs and HEK293 cells exposed to AP or laduviglusib as determined by RT-qPCR (each group *n* = 3) and immunoblotting with an anti-Snail1 antibody. (B) AP and laduviglusib increased the expression of SLC19A3 in HeLa cells (*n* = 3). (C) AP and enforced expression of Snail1 reduced the luciferase activity by a reporter containing the upstream sequence of SLC19A3 gene (*n* = 3). (D) Silencing Snail1 increased SLC19A3 expression and substantially blocked its suppression by AP (n = 3). Data are expressed as mean ± SEM. ^∗^*p* < 0.05, ^∗∗^*p* < 0.01, ^∗∗∗^*p* < 0.001.

It has been shown that GSK3β pathway controls the expression of Snail1, a zinc finger transcription inhibitor that affects the differentiation of stem cells as well as epithelial-mesenchymal transition (EMT).^31^ We asked whether AP and GSK3β inhibition alter the expression of Snail1. As shown in Figure 6A, while induction of ADSCs toward adipocytes reduced the mRNA level of Snail1, AP increased its expression markedly and restored the decreased Snail1 in induced cells, indicating that Snail1 could be a potential molecule that mediates the effects of AP. We then examined the expression of Snail1 in HEK293 cells. These cells expressed relatively low level of Snail1, which was upregulated dose-dependently by AP both at mRNA and protein levels. Further, the level of Snail1 was also increased in the presence of the GSK3β inhibitor laduviglusib.

Previous experiments have indicated, that downregulation of SLC19A3 mediated the inhibition of AP on adipogenesis. Due to the technique challenges in inducing and transfecting ADSCs, we assessed the expression of SLC19A3 in HEK293 cells. Similar to the findings in ADSCs, the level of SLC19A3 was reduced by AP and laduviglusib dose-dependently (Figure 6B). A luciferase reporter containing the 2000 bp upstream element of SLC19A3 gene was then constructed. When transfected into the cells, both co-transfection of Snail1 and exposure to AP reduced the luciferase activity markedly (Figure 6C), suggesting that Snail1 may mediate the downregulation of SLC19A3. Use was then made of siRNA against Snail1. As shown in Figure 6D, knock down of Snail1 upregulated SLC19A3 and largely prevented the downregulation by AP. Taken together, these data indicate that increased Snail1 induced by AP contributes to the downregulation of SLC19A3, which in turn leads to the inhibition of adipogenic differentiation of ADSCs.

## Discussion

Natural products have been an irreplaceable source of drug discovery. Despite the emphasis on high-throughput screening of synthetic libraries, computational molecular design, and more recently development of antibody-based therapeutics, a significant portion of the FDA-approved drugs over the past 30 years are based on natural products or their derivatives.^32^ Andrographolide is one of the main active components from *Andrographis paniculata*, which has been used in traditional medicines in India, China and south Asian countries for centuries.^33^ This diterpene lactone exhibits a wide range of biological activities including antioxidant, anti-inflammatory, antibacterial, immunomodulatory, cytotoxic, neuroprotective, hepatoprotective, and anti-diabetic potential.^34^ Both extracts of *Andrographis paniculata* and a number of formulated andrographolides have been widely used as an anti-inflammatory prescription with a relatively safe record.^6^ However, their low bio-availability and uncertain mechanisms of action remain major hurdles for their wide applications.

Studies in different cells and inflammation models found that inhibition of NF-κB activation, changes in PI3K and MAPK pathways, blocking NFAT activation, and suppression of Keap1-Nrf2 and NLPR3 are involved, respectively, in the anti-inflammatory action of AP.^35^ Thus, it has been proposed that the Michael addition acceptor of AP enable it to react with multiple target proteins and exert the anti-inflammation effect. However, it is also conceivable that AP could act on a multi-functional protein, leading to multiple changes in signaling molecules and biological effects. We found in this study through CETSA that AP likely acts on GSK3β and modulates its function. In cells, GSK3β interacts with hundreds of proteins and has profound effects on inflammations, which was illustrated by the finding that the embryonic lethality of GSK3β deficiency can be rescued by an anti-TNF-α antibody.^36^ Therefore, it is worthy to further examine how the anti-inflammatory actions of AP were mediated by its interaction with GSK3β.

Obesity is an increasing global epidemic that poses a significant challenge for the healthcare system. It is among the highest risk factors for the development of various diseases, including diabetes and cardiovascular diseases, and increased disability and mortality associated with those diseases.^37^^,^^38^ Driven by a combination of genetic, physiological, environmental, and behavioral factors, obesity may result from enlargement of existing adipocytes (hypertrophy) and increase in the number of adipocytes (hyperplasia), which is presumably due to the increased differentiation of adipose stem cells toward adipocytes.^39^ It has been estimated that ADSCs account for a large percentage of fat tissues, ranging from 1% to 10% according to different studies.^40^^,^^41^ In the present study, we found that AP is a potent inhibitor of adipogenesis in cells and in animal fed with HFD. Further investigation revealed the downregulation of thiamine transporter SLC19A3 mediates the anti-differentiation effect of AP. Of note, fat tissue has the highest level of SLC19A3 mRNA among human organs and tissues. In adipocytes, thiamine acts as a key coenzyme of a number of important enzymes, including pyruvate dehydrogenase that converts pyruvate to generate ATP and to support fatty acid synthesis.^42^ Thus, the anti-obesity action of AP in mice may reflect both inhibition of differentiation and suppression of adipogenesis. It is conceivable that AP may represent a novel strategy for weight reduction.

### Limitations of the study

The major limitations of the study at present include.

1) We have not demonstrated undoubtedly through co-crystallization that AP binds directly to GSK3β.
2) We have not proved through genetic studies that GSK3β inhibition is indeed required the action of AP.

## Resource availability

### Lead contact

Further information and requests for resources and reagents should be directed to and will be fulfilled by the lead contact, Yili Yang (nathanyang@icgeb.cn).

### Materials availability

Cell lines and antibodies generated in this study will be available on request through the completion of a material transfer agreement.

### Data and code availability

• All data reported in this article will be shared by the lead contact upon request.
• This article does not report the original code.
• Any additional information required to reanalyze the data reported in this article is available from the lead contact upon request.

## Acknowledgments

This project was initiated in Suzhou Institute of Systems Medicine, and supported by grants from 10.13039/501100001809National Natural Science Foundation of China (10.13039/501100001809NSFC, 81572378, 81973358) and 10.13039/501100002949Jiangsu Province (BZ2022058).

## Author contributions

Y.Y. designed, supported, and supervised the study and prepared the manuscript. X.W. helped prepare the manuscript and performed the studies on Snail1. Y.H. performed the screening and identification of SLC19A3; X.H. and Y.F. carried out the animal studies and started the CESA; G.Z. completed the CESA and thiamine supplementary experiments; Y.J. contributed on the study on Snail1; Y.G. and J.Y. edited the manuscript and supervised the experiments. J.Y. also participated the preparation of ADSCs.

## Declaration of interests

The authors declare no competing interests.

## STAR★Methods

### Key resources table

**Table:** 

REAGENT or RESOURCE	SOURCE	IDENTIFIER
**Antibodies**		

Rabbit monoclonal anti-FABP4	Cell Signaling Technology	Cat#2120S; RRID: AB_2102466
Rabbit monoclonal anti-PPARγ	Cell Signaling Technology	Cat#2435S; RRID:AB_2166051
Rabbit monoclonal anti-SLC19A3	Abcam	Cat# ab237630
Phospho-GSK3β (Ser9) (5B3)	Cell Signaling Technology	Cat#9323S; RRID: AB_2115201
Rabbit polyclonal antibody to GSK3 beta	Affinity Biosciences	Cat#AF5016; RRID: AB_2834935
Phospho-β-Catenin (Ser33/37/Thr41) Antibody	Cell Signaling Technology	Cat#9561S; RRID: AB_331729
Rabbit anti-β-catenin	Cell Signaling Technology	Cat#9562S; RRID: AB_331149
Rabbit anti-Snail (C15D3)	Cell Signaling Technology	Cat#3879S; RRID: AB_2255011
Rabbit polyclonal anti-GFP	MBL International	Cat#598, RRID: AB_591819
APC anti-human CD34 antibody	BioLegend	Cat#378605, RRID: AB_3068132
PE anti-human CD73 antibody	BioLegend	Cat#344003, RRID: AB_2298698
PE anti-human CD105 antibody	BioLegend	Cat#323205, RRID: AB_755957
Mouse monoclonal anti-β-Actin	TransGen Biotech	Cat#HC201; RRID:AB_2860007
Mouse monoclonal anti-GAPDH	TransGen Biotech	Cat#HC301; RRID:AB_2629434
HRP-labeled Goat Anti-Rabbit IgG(H+L)	Beyotime Biotechnology	Cat#A0208
HRP-labeled Goat Anti-Mouse IgG(H+L)	Beyotime Biotechnology	Cat#A0216
FITC-labeled Goat Anti-Rabbit IgG (H+L)	Beyotime Biotechnology	Cat#A0562

**Chemicals, peptides, and recombinant proteins**		

Natural Product Library	Selleck Chemicals	Cat#L1400
Andrographolide	MedChemExpress (MCE)	Cat#HY-N0191
Laduviglusib	MedChemExpress (MCE)	Cat#HY-10182
Collagenase, Type I	MedChemExpress (MCE)	Cat#HY-E70005A
Oil red O staining solution	Sigma-Aldrich	Cat#1.02419
Insulin	Sigma-Aldrich	Cat#I9278
Dexamethasone	Sigma-Aldrich	Cat#D4902
Indomethacin	Sigma-Aldrich	Cat#I7378
3-Isobutyl-1-methylxanthine (IBMX)	Sigma-Aldrich	Cat#I5879
Thiamine (Vitamin B1)	Sigma-Aldrich	Cat#T1270
Sodium carboxymethyl cellulose (CMC)	Sigma-Aldrich	Cat#419273
β-Glycerophosphate	Beyotime Biotechnology	Cat#ST637

**Critical commercial assays**		

Lipofectamine™ RNAiMAX Transfection Reagent	Thermo Fisher Scientific	Cat#13778150
Cell Counting Kit-8 (CCK-8)	MedChemExpress (MCE)	Cat#HY-K0301
TransScript® All-in-One First-Strand cDNA Synthesis SuperMix for qPCR	TransGen Biotech	Cat#AT341-02
NEBNext® Ultra™ RNA Library Prep Kit for Illumina®	New England Biolabs	Cat#E7530S
RNAiso Plus Reagent	Takara	Cat#9108
Immunohistochemistry Kit (Goat Anti-Rabbit IgG H&L (HRP))	Wuhan Servicebio Technology	Cat#G1215

**Deposited data**		

mRNA sequencing data	This paper	N/A

**Experimental models: Cell lines**		

Human adipose-derived stem cells (hADSCs)	This paper	N/A
3T3-L1	Cellosaurus	Cat#CL-173; RRID: CVCL_0123
HEK293T	National Collection of Authenticated Cell Cultures	Cat#GNHu44

**Experimental models: Organisms/strains**		

C57BL/6 mice	Shanghai Model Organisms Center, Inc.	Cat#SM-001

**Oligonucleotides**		

RT-qPCR primers	GENEWIZ Suzhou, China	See Table S2
siRNA oligonucleotides	Suzhou GenePharma Co., Ltd.	See Table S2

**Software and algorithms**		

FlowJo™ v10 software	BD Biosciences	N/A
ImageJ	National Institutes of Health	N/A
Prism 8	GraphPad	N/A
BioRender	BioRender	https://www.biorender.com/

### Experimental model and study participant details

#### Human adipose-derived stem cells (hADSCs)

Human adipose-derived stem cells (hADSCs) were isolated from the adipose tissues of obese patients between 20 and 35 years old undergoing surgery treatments for obesity. All the patients signed agreements for the isolation of their adipose tissues for research purposes in accordance with the Guidelines of Research Application of Clinical Samples set by Soochow University Affiliated First Hospital. After surgery, adipose tissues were immediately transported to the laboratory in sterile 1 x PBS containing 100 U/ml penicillin and 100 μg/ml streptomycin on ice. After washing 2 times with PBS, the adipose tissues were minced by using sterile scalpels and scissors and then digested with 0.1% type I collagenase in a shaking water bath at 37°C for 30 min. After digestion, tissues were filtered with 70 μm mesh sieves, and the filtrates were centrifuged at 1000 g for 10 min at room temperature. Cells obtained from the pellet were cultured with DMEM/F-12 containing 10% FBS and antibiotics.

#### Mice

Male 12-week-old wild-type (WT), ob/ob, and db/db C57BL/6 mice were purchased from Shanghai Laboratory Animal Center (SLAC). Animal experiments were conducted following protocols approved by the Institutional Animal Care and Use Committee (IACUC) of Suzhou Institute of Systems Medicine. Mice were housed in pathogen-free (SPF) facility under a 12-hour light/dark cycle. 8-week-old male C57BL/6 WT mice were fed either normal chow diet (NC) or high-fat diet (HFD) for 2 months to induce obesity. Andrographolide (AP) was administered in 0.5% carboxymethyl cellulose (CMC, Sigma-Aldrich), starting either at the onset of the HFD feeding or after 4 weeks. Body weight and food intake were monitored weekly. At the end of the treatment period, mice were sacrificed, and adipose tissues, along with other organs, were harvested for experimental analyses.

### Method details

#### Phenotypical analyses of hADSCs

The expression of specific surface markers of ADSCs was evaluated by flow cytometry by using BD FACSCalibur™ (Becton Dickinson, USA). After trypsinization, cells were suspended in 1 x PBS and incubated for 30 min at 4°C in dark with anti-human CD34, CD73, CD105 monoclonal antibodies conjugated with fluorophores (Biolegend, USA). After that, cell pellets were washed twice with 1 x PBS. After the second wash, cell pellets were fixed with 1% (w/v) paraformaldehyde (PFA) in PBS. BD FACSuite™ software was used to collect and analyze at least 10,000 labeled cells. Adipose cells were sorted as CD34^−^/CD73^+^/CD105^+^ by flow cytometry using BD FACSAria™ II Cell Sorter. After 3 passages, the cells were frozen in liquid nitrogen for further experiments.

#### Analyses of adipogenic differentiation of hADSCs

To evaluate the capabilities of hADSCs differentiating into adipocytes, ADSCs were seeded at 5 × 10^4^ cells/cm^2^ density into each well of a six-well plate with regular growth medium. After reaching 70-80% confluence, the cells were cultured with adipogenic induction medium comprising DMEM, 10% FBS, 10 μg/mL insulin, 0.5 mM isobutylmethylxanthine (IBMX), 1 μM dexamethasone, and 200 μM indomethacin. Control cells were cultured in regular growth medium. After 14 days, cells were fixed with 4% PFA and stained with 0.5% Oil Red O staining solution for 10 min and examined under an inverted phase contrast microscope (Nikon TS100, Japan) for the accumulation of lipid droplets in adipocytes. To quantify intracellular triglycerides, cells were washed with PBS and incubated with 500 μL of isopropyl alcohol, and analyzed by a spectrophotometer with an excitation wavelength at 510 nm to obtain quantitative data.

#### Screening of natural compounds

A natural product library comprising 124 compounds was screened to identify inhibitors of adipogenic differentiation. ADSCs were seeded in 96-well plates at a density of 5 × 10^3^ cells per well and cultured in adipogenic induction medium. Compounds were added at a final concentration of 10 μM or 0.1% DMSO as controls. After 7 days, total RNAs were extracted using TRIzol (Takara, Japan), and the expression of adipogenic markers LPL and FABP4 was quantified by RT-qPCR, with GAPDH used as internal control. Compounds that significantly inhibited both LPL and FABP4 expressions were identified as potential inhibitors of adipogenic differentiation.

#### Cell viability assay (CCK-8)

Cell viability was measured using the Cell Counting Kit-8 (CCK-8). ADSCs were plated at a density of 5 × 10^3^ cells per well in 96-well plates containing 100 μL of media. Cells were treated with indicated concentrations of andrographolide (AP) for 24 hours. CCK-8 was then added to each well and incubated for 2 hours. Absorbance was measured at 450 nm using a microplate reader to quantify cell viability.

#### Reverse-transcription quantitative PCR (RT-qPCR)

Total RNAs were extracted using Trizol (Takara) reagent according to the manufacturer’s protocol, and the concentrations of total RNAs were measured with Nanodrop-ND1000 (Thermo Fisher Scientific, Waltham, MA). 500 ng of each total RNA was used for reverse transcription to synthesize cDNA with the TransScript® cDNA synthesis kit (TransGen Biotech). Exact same amounts of cDNAs were used for qPCR amplifications with a SYBR Green SuperMix (TransGen Biotech). The amplifications and quantifications of relative gene expressions were carried out with the LightCycler® 480 System (Roche). Primers used for qPCR amplifications are listed in Table S2.

#### RNA sequencing (RNA-seq)

Human adipose-derived stem cells were cultured in adipogenic-induction medium for 3 days to induce adipogenic differentiation. Total RNAs were extracted from both treated and untreated cells using the TRIzol reagent (Takara) as described above. RNA samples with RNA integrity number (RIN) values greater than 7 and 1 μg RNA quantity were used to prepare libraries for next-generation sequencing. Library preparation was performed according to the manufacturer’s protocol using the NEBNext® Ultra™ RNA Library Prep Kit for Illumina®. The resulting sequences were processed and analyzed by GENEWIZ (Suzhou, China).

#### Immunoblotting

ADSCs were cultured in 10-cm dishes and treated with different concentrations of AP for 7 days. Cells were washed once with PBS and then lysed on ice for 20 min with RIPA buffer (Beyotime, P0013C) supplemented with protease inhibitor cocktail (MedChemExpress, HY-K0010). Protein concentrations were measured using the bicinchoninic acid (BCA) assay (Thermo Scientific, Rockford, IL, USA). 30 μg total proteins of each sample were electrophoresed in an SDS–PAGE gel and transferred onto PVDF membranes (Whatman, Dassel, Germany). After blocking with 3% BSA for 1 hr at room temperature, the membranes were incubated overnight at 4°C with specific primary antibodies. After that, the membranes were washed 3 times with 1x TBST, followed by incubation with HRP-conjugated secondary antibodies for 1 hr at room temperature. Finally, the membranes were washed 3 times with 1× TBST for a total of 30 min, followed by blotting with ChemiDoc™ XRS+ Imaging Systems with Image Lab™ Software (Bio-Rad). Quantification of certain blots was carried out with ImageJ software. Antibodies used in this study are shown in key resources table.

#### Cellular thermal shift assay (CETSA)

The cellular thermal shift assay (CETSA) was performed according to the method described by Martinez Molina et al. Briefly, ADSCs suspended in PBS containing protease inhibitors were aliquoted into PCR tubes and heated at the indicated temperatures in the presence or absence of andrographolide. After heating for 3 minutes, tubes were cooled to room temperature for 3 minutes. Cells were then lysed by 2 freeze-thaw cycles with liquid nitrogen and a heating block set at 25°C. Cell lysates were then centrifuged at 20,000 x g for 20 minutes at 4°C. The supernatants were collected and analyzed by immunoblotting with anti-GSK3β antibody.

#### Dual-luciferase reporter assay

For the reporter assays, 293T cells were seeded at a density of 1 × 105 cells per well in a 24-well plate and cultured overnight. The cells were co-transfected with 400 ng of SLC19A3-luc reporter construct and 4 ng of Renilla construct using Lipofectamine 3000 (Life Technologies) according to the manufacturer’s instructions. Twenty-four hours after the transfection, the cells were treated with different concentrations of AP for 24 hours. Then the cells were lysed in 100 μL of passive lysis buffer, and the lysates were assayed for luciferase activity using the Dual-Luciferase Assay System (Promega) on a plate reader (Corning). All experiments were performed in triplicate and data were expressed as mean±SD.

### Quantification and statistical analysis

Band intensities of immunoblots in this study were quantified using the software ImageJ (National Institutes of Health). Data were analyzed using GraphPad Prism 9.0 (GraphPad Software Inc., San Diego, CA, USA) with one-way ANOVA and Newman–Keuls multiple comparison tests. Results are expressed as mean ± SEM unless otherwise indicated. All P values less than 0.05 were considered as statistically significant.

## Supplemental information

Document S1. Figures S1–S3

Table S1. Chemical compounds of the natural product library

Table S2. List of all primers used in this study and their sequences
