# Supplementary material for: Andrographolide inhibits the upregulation of SLC19A3 to block the adipogenic differentiation of adipose-derived stem cells
Source: iScience. 2025 Dec 8;29(1):114379. doi: 10.1016/j.isci.2025.114379 (PMC12794426; doi:10.1016/j.isci.2025.114379)
Supplement: Document S1. Figures S1–S3 [file mmc1.pdf]

iScience, Volume *29*

Supplemental information

Andrographolide inhibits the upregulation of SLC19A3 to block the adipogenic differentiation of adipose-derived stem cells

Yili Yang, Xuan Wang, Guangfeng Zhou, Xiaodan Hou, Yang He, Yuan Feng, Yuxue Jiang, Yuexi Gu, and Jun Ye

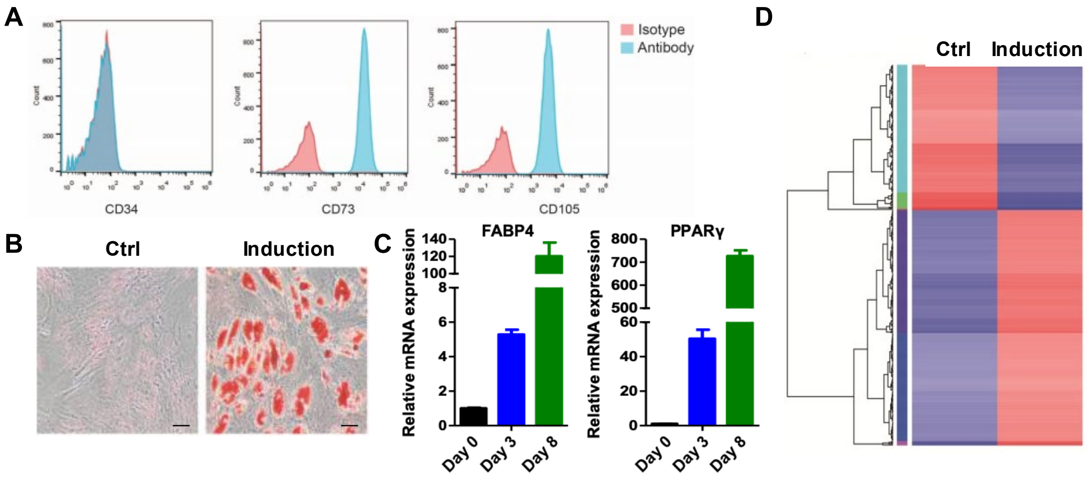

Supplement Figure 1. Adipogenic differentiation of adipose-derived stem cells (ADSCs). **A.** Phenotypes of ADSCs; **B, C, D.** Induction of differentiation of ADSCs revealed by oil red O staining (Scale bars, 100 μm) (B), expression of markers assessed by RT-qPCR (C), and RNA-seq analysis (D).

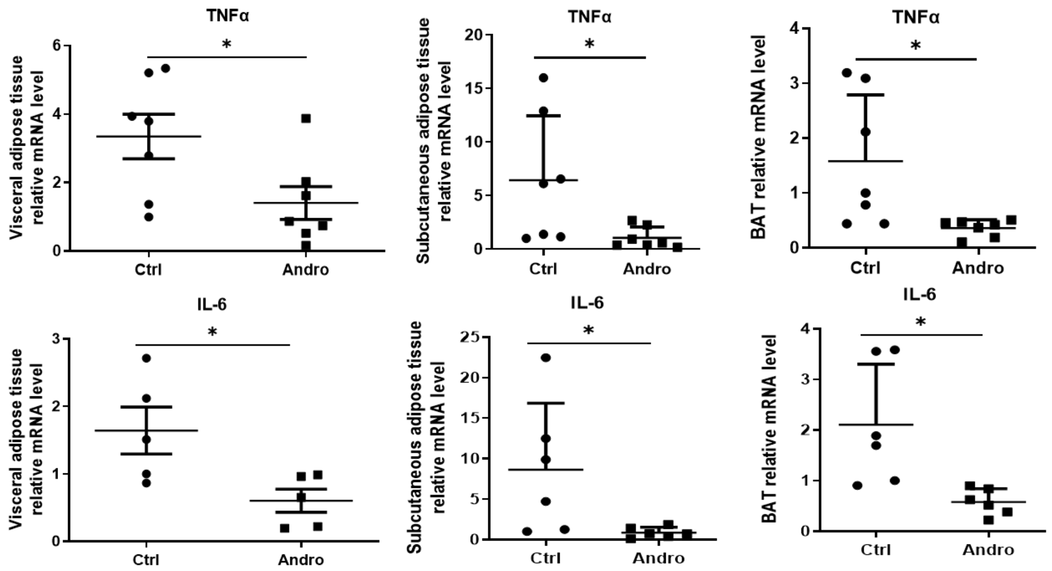

Supplement Figure 2. Andrographolide reduces the levels of inflammatory cytokines TNFα and IL-6 in mice fed with high fat diet in visceral, subcutaneous, and brown adipose tissues (BAT). Data are expressed as mean ± SEM. $^{*}p<0.05$.

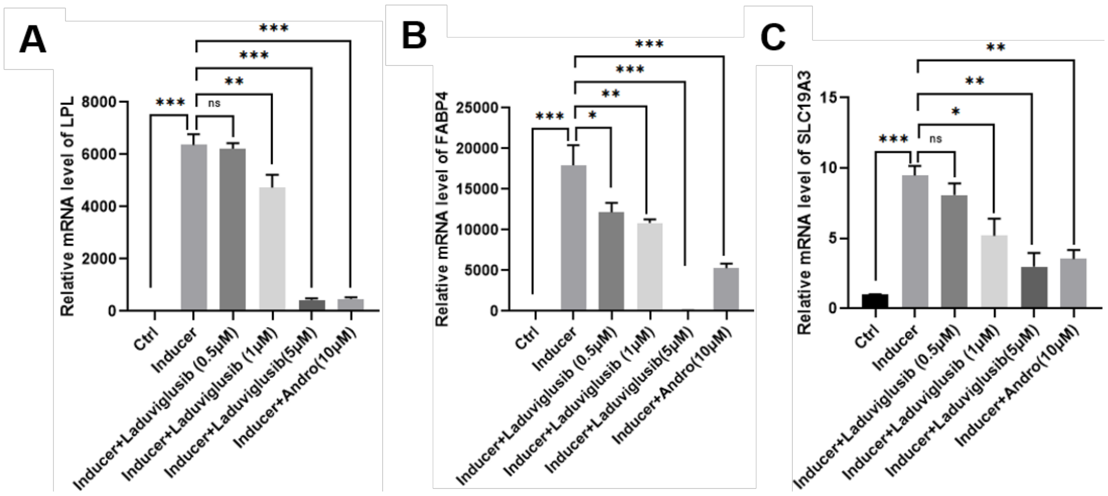

Supplement Figure 3. GSK3β inhibitor laduviglusib inhibited the expression of adipogenic differentiation markers LPL (**A**), FABP4 (**B**), and SLC19A3(**C**). Data are expressed as mean ± SEM. $^{*}p$<0.05, $^{**}p$<0.01, $^{***}p$<0.001.
